# Roles of the *SPL* gene family and miR156 in the salt stress responses of tamarisk (*Tamarix chinensis*)

**DOI:** 10.1186/s12870-019-1977-6

**Published:** 2019-08-22

**Authors:** Jianwen Wang, Youju Ye, Meng Xu, Liguo Feng, Li-an Xu

**Affiliations:** 1 Key Laboratory of Forestry Genetics & Biotechnology of Ministry of Education, Co-Innovation Center for Sustainable Forestry in Southern China, Nanjing Forestry University, Nanjing, 210037 China; 2 College of Horticulture and Plant Protection, Yangzhou University, Yangzhou, 225009 China

**Keywords:** *SPL* gene family, miR156, Salt stress, *Tamarix chinensis*

## Abstract

**Background:**

Accumulating evidences show that *SPL*s are crucial regulators of plant abiotic stress tolerance and the highly conserved module miR156/*SPL* appears to balance plant growth and stress responses. The halophyte *Tamarix chinensis* is highly resistant to salt tress. *SPL*s of *T. chinensis* (*TcSPLs*) and theirs roles in salt stress responses remain elusive.

**Results:**

In this study, we conducted a systematic analysis of the *TcSPLs* gene family including 12 members belonging to 7 groups. The physicochemical properties and conserved motifs showed divergence among groups and similarity in each group. The microRNA response elements (MREs) are conserved in location and sequence, with the exception of first MRE within *TcSPL*5. The miR156-targeted *SPL*s are identified by dual-luciferase reporter assay of MRE-miR156 interaction. The digital expression gene profiles cluster suggested potential different functions of miR156-targeted *SPL*s vs non-targeted *SPL*s in response to salt stress. The expression patterns analysis of miR156-targeted *SPL*s with a reverse expression trend to TcmiR156 suggested 1 h (salt stress time) could be a critical time point of post-transcription regulation in salt stress responses.

**Conclusions:**

Our work demonstrated the post-transcription regulation of miR156-targeted *TcSPLs* and transcription regulation of non-targeted *TcSPLs* in salt stress responses, and would be helpful to expound the miR156/*SPL*-mediated molecular mechanisms underlying *T. chinensis* salt stress tolerance.

**Electronic supplementary material:**

The online version of this article (10.1186/s12870-019-1977-6) contains supplementary material, which is available to authorized users.

## Background

Transcription factors (TFs) are proteins binding to cis-acting elements of target genes to activate or inhibit their transcription [1]. Dozens of plant TF gene families precisely coordinate the spatial and temporal expression of downstream genes associated with abiotic stress, such as AP2 (APETALA2), NAC (NAM, ATAF1/2, and CUC2), auxin response factors (ARFs), and SQUAMOSA promoter-binding (SBP) protein-like proteins (SPLs) [2–5]. SPLs are plant-specific TFs that contain a highly conserved DNA binding domain SBP-box consisting of two zinc finger structures [6]. The essential roles of SPLs in plant development, including shoot and leaf morphogenesis, floral organ development, flowering and fruit ripening, have been thoroughly established [7, 8].

Recent studies indicate that *SPL* genes are crucial regulators of abiotic stress tolerance in many plants [9–11]. Genome-wide profile studies of the *SPL* family in plant species have found many *SPL* responses to salt, drought, cold and heat stress [12–14]. Some of these *SPL*s participate in salt/drought stress responses by regulating the abundance of genes involved in signal transduction, reactive oxygen species (ROS) scavenging, accumulation of POD/SOD, proline synthesis and anthocyanin metabolism. For instance, *VpSBP16* of *Vitis pseudoreticulata* enhances the salt and drought stress tolerance of overexpressed *Arabidopsis* by regulating the salt overly sensitive (SOS) and ROS signalling [10]. *BpSPL9* confers tolerance to salt and drought stress upon *Betula platyphylla* by improving scavenging of ROS [15].

Furthermore, a subset of *SPL*s with miRNA response element (MRE) complementary to miR156 are regulated by miR156 mediated mRNA cleavage and/or translational repression at the post-transcriptional level [16, 17]. For example, *SPL13* silencing caused by miRNA156 improves the drought stress tolerance of alfalfa [18]. The miR156/*SPL* studies increasingly illuminate that the miR156/*SPL* module emerges as a key bridge to the balance of plant abiotic stress responses and development [9, 11]. In *Arabidopsis* and rice, miR156 overexpression increased salt stress tolerance and delayed flowering. The miR156 knock-down lines and *SPL9* overexpression lines showed the opposite phenotype (sensitivity to salt stress and early flowering) by anthocyanin metabolism regulation. Similar results were identified in the miR156/*SPL3* study on flowering timing and hot stress memory in *Arabidopsis*. Accumulating evidence shows that the miR156/*SPL* module is highly conserved in land plants and appears to be useful molecular tools in plant growth (biomass/yield/flowing time) and stress resistance improvement [19, 20]. As a typical case, *OsSPL14* and *OsSPL16* in rice improve grain quality and yield as well as *SPL* homologues in maize do [21, 22]. Moreover, downregulation of *SPL8* improved both the biomass yield and the salt/drought tolerance of transgenic alfalfa, indicating that miR156-targeted *SPL8* has considerable potential in legume breeding [23].

*Tamarix chinensis* (*T. chinensis*), as a halophyte that is highly resistant to salinity, is a suitable material for investigating plant salt stress tolerance [24, 25]. Although many TF genes that are associated with salt or drought stress have been identified in *Tamarix spp*. [26–31], the *SPL*s of *T. chinensis* (*TcSPLs*) and their roles in salt stress responses remain to be elucidated. In this study, systematic identification of the *TcSPL* family with classification, structure analysis and expression profile analysis provided sequence characterization of *TcSPLs* and expression patterns under salt stress. Furthermore, we conducted a preliminary investigation of the expression patterns of miR156-targeted *SPL*s to determine their roles in salt stress responses by experimental verification.

## Results

### Identification of *TcSPL* genes and miR156 targets

To further explore the important role of the *SPL* genes in *T. chinensis* (*TcSPLs*) under salt stress, the cDNA sequences of the *TcSPL* family were predicted from the RNA-seq data [32]. All 14 *TcSPLs*, except *TcSPL13* and *TcSPL14*, contained conserved SBP-boxes (Table 1). Six members of the *TcSPL* family containing miRNA response elements (MRE) are potential targets of TcmiR156 (Table 2). It is notable that two MREs are predicted in *TcSPL5,* including a mRNA cleavage site and a translation inhibition site.

**Table 1:** Identification of Squamosa promoter-binding protein-like (*SPL*) in *Tamarix chinensis*

Gene	RNA-seq ID	Source	CDS (bp)	SBP-box	MRE type	MRE location
*TcSPL1*	comp24748	RACE	1449	conserved	cleavage	CDS
*TcSPL2*	comp17803	RACE	624	conserved	cleavage	3’UTR
*TcSPL3*	comp19945	RACE	1359	conserved	cleavage	CDS
*TcSPL4*	comp14758	RACE	576	conserved	cleavage	3’UTR
*TcSPL5*	comp16909	RACE	1338	conserved	inhibition; cleavage	CDS
*TcSPL6*	comp8034	PCR	3024	conserved	\	\
*TcSPL7*	comp25771	PCR	2502	conserved	\	\
*TcSPL8*	comp16485	PCR	1203	conserved	cleavage	CDS
*TcSPL9*	comp19275	PCR	2043	conserved	\	\
*TcSPL10*	comp20377	PCR	1743	conserved	\	\
*TcSPL11*	comp87279	Predicted	666^a^	conserved	\	\
*TcSPL12*	comp9552	Predicted	492^a^	conserved	\	\
*TcSPL13*	comp172543	Predicted	309^a^	Incomplete	\	\
*TcSPL14*	comp15035	Predicted	312^a^	Incomplete	\	\

^a^means partial CDS

**Table 2:** Protein feature of Squamosa promoter-binding protein-like family (*SPL*) in phylogenetic analysis

Group	SPL family	Len (AA)	pI	Mw (KDa)	MRE
I	*TcSPL7*	833	6.34	93.7	\
	*AtSPL7*	818	6.5	91.6	\
	*PtSPL3*	738	6.8	83.0	\
	*PtSPL4*	793	6.4	88.9	\
IIa	*TcSPL6*	1007	6.59	111.4	\
	*TcSPL9*	1098	7.70	120.7	\
	*TcSPL10*	989	6.76	109.7	\
	*AtSPL1*	881	5.6	98.5	\
	*AtSPL12*	927	5.9	104.1	\
	*AtSPL14*	1035	8.7	114.8	\
	*AtSPL16*	988	8.3	89.6	\
	*PtSPL1*	1030	8.0	114.8	\
	*PtSPL2*	1044	8.3	116.1	\
	*PtSPL5*	1035	7.3	115.4	\
	*PtSPL6*	1004	6.1	111.3	\
	*PtSPL7*	1002	5.9	110.9	\
	*PtSPL9*	1039	8.0	115.0	\
IIb	*TcSPL12*	^a^	^a^	^a^	^a^
	*AtSPL8*	333.0	9.0	36.8	\
	*PtSPL21*	325	9.1	36.1	\
	*PtSPL26*	328	8.9	36.4	\
IIc	*TcSPL11*	^a^	^a^	^a^	^a^
	*AtSPL13A*	359	8.0	39.1	miR156
	*AtSPL13B*	359	7.9	39.1	miR156
	*PtSPL14*	381	8.7	41.8	miR156
	*PtSPL15*	346	8.7	38.3	miR156
	*PtSPL18*	376	9.3	41.3	miR156
	*PtSPL22*	313	9.23	34.2	miR156
IId	*TcSPL*2	207	9.84	22.8	miR156
	*TcSPL*4	191	9.36	21.7	miR156
	*AtSPL*3	131	8.2	15.3	miR156
	*AtSPL*4	174	9.7	20.1	miR156
	*AtSPL*5	181	9.8	21.0	miR156
	*PtSPL*16	144	6.5	16.2	miR156
	*PtSPL*20	196	9.2	22.0	miR156
	*PtSPL*23	148	7.6	16.5	miR156
	*PtSPL*24	202	9.5	22.7	miR156
	*PtSPL*25	193	9.1	21.7	miR156
IIe	*TcSPL*3	452	7.25	50.2	miR156
	*TcSPL*5	445	6.28	49.3	miR156
	*TcSPL*8	400	8.60	41.6	miR156
	*AtSPL*6	406	7.6	45.4	miR156
	*AtSPL*9	375	8.4	40.8	miR156
	*AtSPL*15	354	9.1	39.7	miR156
	*PtSPL*8	398	8.5	43.8	miR156
	*PtSPL*12	510	7.3	55.9	miR156
	*PtSPL*13	600	8.0	66.2	miR156
	*PtSPL*27	408	6.8	45.2	miR156
	*PtSPL*28	561	8.4	61.6	miR156
	*PtSPL*17	375	8.9	40.2	miR156
IIf	*TcSPL*1	482	9.23	52.5	miR156
	*AtSPL*2	419	8.9	46.9	miR156
	*AtSPL*10	396	7.9	44.2	miR156
	*AtSPL*11	393	8.4	43.9	miR156
	*PtSPL*11	485	8.9	53.7	\
	*PtSPL*19	435	8.7	47.9	miR156
	*PtSPL*29	472	8.6	51.7	miR156

^a^ mean data missing

The deduced protein length, isoelectric point (pI) and molecular weight of *TcSPL* protein are within a large variation range (Table 2). *TcSPL* proteins ranging from 191 (*TcSPL4*) to 1098 (*TcSPL9*) amino acids had a predicted molecular mass of 21.7 (*TcSPL4*) to 120.7 (*TcSPL9*) kDa, and the pI values ranged from 6.28 (*TcSPL5*) to 9.84 (*TcSPL2*). These results indicated the diversity of the SPL protein family in *T. chinensis*.

### Phylogenetic analysis of *SPL* genes

**Fig. 1:**
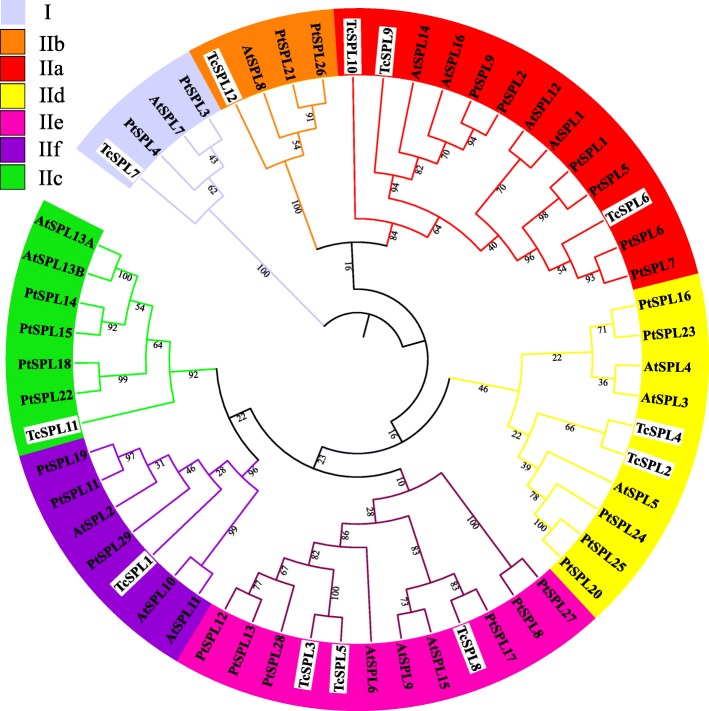
Phylogenetic tree of 59 *SPLs* in *Tamarix chinensis* (*TcSPLs*), *Arabidopsis thaliana (AtSPLs)*, and *Populus trichocarpa (PtSPLs).* The phylogenetic tree was constructed based on the SBP-box. The numbers on the branches indicate the bootstrap value. The branches and groups are distinguished with different colours. *TcSPLs* are emphasized by white background

The *SPL* gene function has been fully clarified in *Arabidopsis and Populous* due to its importance in plant development regulation. To further study the *SPL* evolutionary relationship among *T. chinensis*, *Arabidopsis* (http://planttfdb.cbi.pku.edu.cn/family.php?sp=Ath&fam=SBP), and *Populous trichocarpa* (http://planttfdb.cbi.pku.edu.cn/family.php?sp=Ptr&fam=SBP), the SBP-box sequences of 12 *TcSPLs*, 17 *AtSPLs* from *Arabidopsis* and 30 *PtSPL*s from *P. trichocarpa* were used to construct a maximum likelihood method (ML) tree (Fig. 1 and Table 2). All 59 *SPL* genes were divided into 7 groups containing at least one *SPL* from each species (Fig. 1). The topological structure of ML-tree and corresponding *SPL* members are consistent with multiple *SPL* studies, and the groups were named Group I and IIa-f according to the representative phylogenetic tree of study.

IId includes the smallest *SPL* gene, encoding fewer than 210 amino acids, while members in IIa encode the largest *SPL* proteins with approximately 1000 amino acids. The similar protein feature of *SPL*s in each group indicated that the SBP-box is a relatively conserved domain among all *SPL* genes in different species, and its degree of conservation corresponds to that of *SPL* proteins. In addition, the conserved MRE are identified in all members of IIc-f, except *TcSPL*11 and *PtSPL*11.

### Identification of conserved motifs in *TcSPLs*

**Fig. 2:**
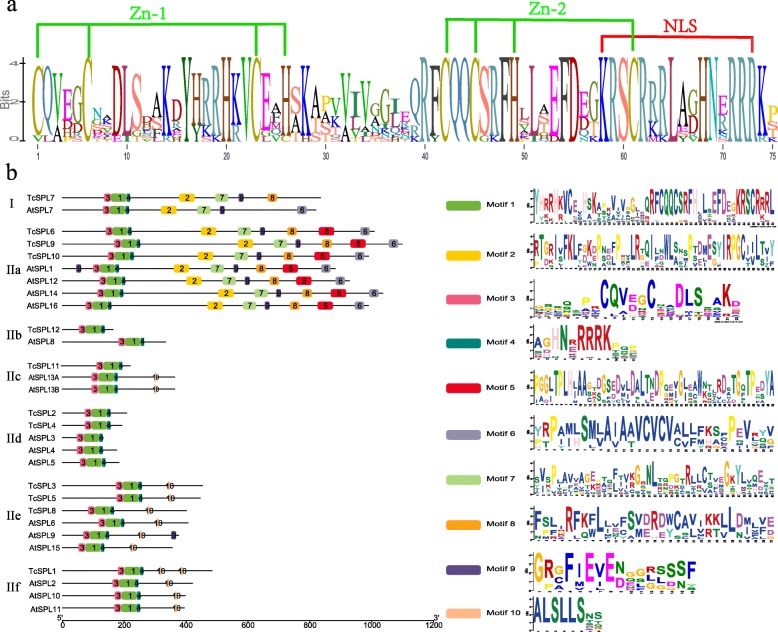
Conserved motif analysis of the *SPL*s of *Tamarix chinensis* (*TcSPLs*). **a** Sequence logo of the SBP-box of *TcSPLs*. The overall height of each stack represents the conservation level of corresponding residue site. The height of the letters within each stack indicates the relative frequency. **b** Distribution of conserved motifs between *TcSPLs* and *AtSPLs* (*SPLs* of *Arabidopsis*). The conserved motifs identified by MEME are represented with boxes of different colours. The numbers 1–10 and corresponding sequence logos represent motifs 1–10, respectively. The sequence logos are based on all *TcSPLs* and *AtSPLs*

The SBP-boxes shared by all *TcSPLs* were predicted by NCBI CD-Search (Conserved Domain Search Service). The sequence alignment (Fig. 2a) showed that the SBP-boxes of *TcSPLs* are conserved structures that contain two zinc finger motifs (Zn-1/2) and a nuclear localization signal (NLS). The Zn-1 motif is a CCCH (C3H)-type zinc finger structure in all *TcSPLs*, except the first Cys residue in *TcSPL*8 substituted with a Val residue, and the CCCC (C4) type in *TcSPL*7. The Zn-2 motif (i.e., C2HC type zinc finger structure) is highly conserved in all *TcSPLs*, which overlap with NLSs with four residues.

The top-8 most conserved motifs and 2 relative conserved motifs predicted in MEME are designated motifs 1 to motif 10 (Fig. 2b). Most of the domains were relatively conserved between *T. chinenses* and *Arabidopsis* [12]. Members of the same group had similar motif compositions. Motifs 3, 1 and 4 represent Zn-1/2 and NLS of SBP-boxes identified in all *TcSPLs*. *TcSPLs* of IIb and IId only contained motifs of the SBP-box. Some motifs were specifically present in one or two groups. Motif 5 was unique to IIa, and Motifs 2, 6, 7 and 8 were unique to I and IIa (Fig. 2b). For the 2 relative motifs, motif 9, including an ankyrin-repeat-containing domain, was found in four *TcSPLs* (*TcSPL*6, − 7, − 9, − 10), indicating that the biological function of these *TcSPLs* may be associated with protein–protein interactions. Furthermore, motif 10 consists of a conserved motif coded by MRE of miR156.

### Digital gene expression profiles of the *TcSPLs* under salt stress

**Fig. 3:**
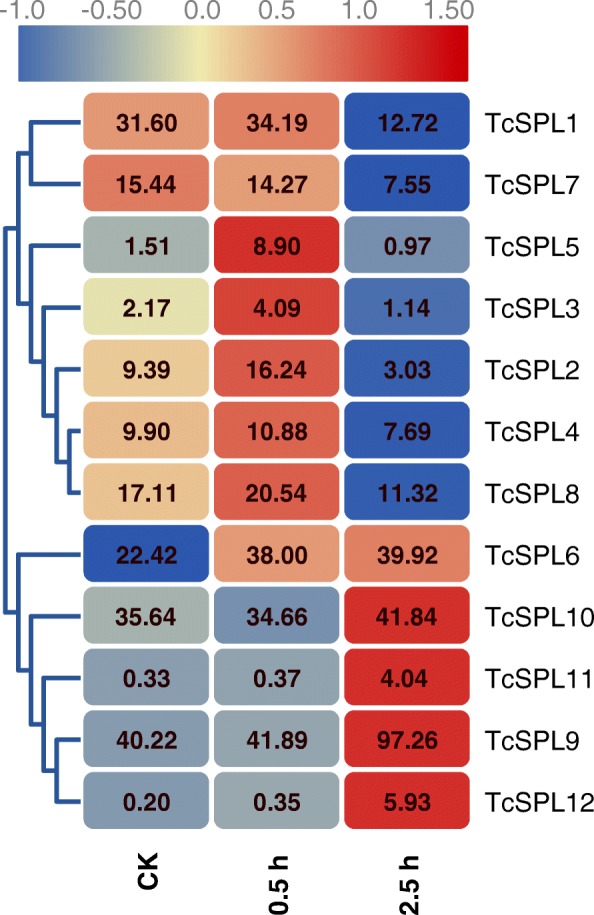
Digital gene expression profiles of the *SPL* genes in *Tamarix chinensis* roots. The heat map was generated based on the normalized Log2 (FPKM) represented by the red-blue gradation. The columns of ck, 0.5 h and 2.5 h represent the 0 h, 0.5 h and 2.5 h NaCl treatments, respectively. The rows represent *SPL* genes. The numbers in the heat map were FPKM from RNA-seq data. The branches represent clusters by complete linkage clustering of Euclidean distance

The phylogenetic and protein motif analyses showed the conservation and diversity of *TcSPLs*. Digital gene expression profiles (DGE) of *TcSPLs* in *T. chinensis* roots were constructed to explore their potential roles in salt stress. Based on clustering of time series consisting of 0, 0.5 h and 2.5 h NaCl treatment, 12 *TcSPLs* were classified into two clusters, i.e., one cluster of downregulated expression pattern and the other cluster of upregulated expression pattern (Fig. 3). All *TcSPLs* with MREs are clustered into the downregulated cluster with the opposite expression pattern of other *TcSPLs* (except *TcSPL*7), implying that TcmiR156-targeted *TcSPLs* may play different roles in *T. chinensis* salt stress responses compared with non-targeted *TcSPLs*. *TcSPL3* and *TcSPL5* of the downregulated cluster showed a significant upregulation in 0.5 h, indicating that the expression patterns of the two *TcSPLs* are complicated. Five *TcSPLs* showed relatively high abundance (FPKM > 20), and three of them (*TcSPL6, − 9, − 10*) belonged to the same group (IIa).

### Validation of TcmiR156-targeted *TcSPLs*

**Fig. 4:**
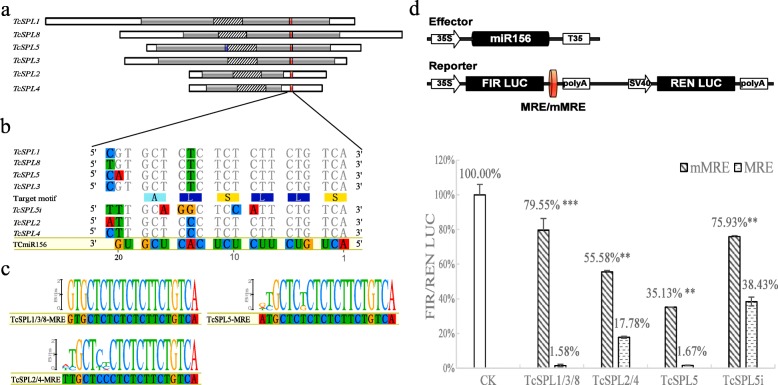
Identification and validation of miR156-targeted *SPL*s of *Tamarix chinensis* (*TcSPLs*). **a** Graphic representation of the cDNAs of all *TcSPLs* carrying the predicted MRE (red and blue boxes). Coding sequences are shaded grey, and the conserved SBP-box is shaded with diagonal. **b** Alignment of the predicted MRE sequences compared with the miR156 reverse complementary sequence (TcmiR156). Nucleotide divergences compared with TcmiR156 are highlighted by colours. Reading frames are indicated by triplet codons, and the target motif is represented by amino acid residues. **c** Sequence logos of MRE within *TcSPLs* and putative *SPL* orthologues of *Arabidopsis thaliana* and *Populus trichocarpa* (*AtSPLs* and *PtSPL*s). *TcSPL*1/3/8-MRE was aligned with, *TcSPL*2/4-MRE was aligned with, *TcSPL*1/3/8-MRE was aligned with, and the *TcSPL5i*-MRE targeted motif was aligned with. **d** Schematic representation of the dual-luciferase reporter (DLR) assay. The reporter and effector were transferred into *Populus* protoplasts after overnight transformation. The ratio of firefly luciferase to Renilla luciferase of four MREs represents the activity of the miR156-*TcSPL* interaction. mMRE are MRE mutated in 10-11th nucleotides of TcmiR156. Data are represented by percentage with SEs (*n* = 4). Significant differences were determined by Student’s t-test (**P < 0.01, *** *P* < 0.001)

Six *TcSPLs* targeted by TcmiR156 contain 7 MREs of the *SPL* mRNAs: 6 mRNA cleavage MREs (*SPL*-MREs) and one translation inhibition MRE (*TcSPL5i*-MRE). All *SPL*-MREs lie downstream of the conserved SBP-box and are part of the coding sequence (CDS) of the last exon (Additional file 3: Figure S1) with the exception of *TcSPL2*-MRE and *TcSPL4*-MRE located in the 3′ untranslated region (UTR). Unlike *SPLc*-MREs, *TcSPL5i*-MRE is located in the first exon and overlaps with 4 upstream nucleotides of the SBP-box (Fig. 4a). The alignments of the TcmiR156 reverse complement with *SPL*-MREs (Fig. 4b) show the perfect complementarity of *TcSPL*-MREs to 5′ of TcmiR156 and two mismatches to 3′ of TcmiR156 (the 14th and last nucleotides). In addition to the 14th and last nucleotides, four mismatches (the 9th, 10th, 15th and 16th nucleotides) with TcmiR156 indicated that *TcSPL*5 could be translationally inhibited by miR156 in *TcSPL5i*-MRE. The miR156-related MREs except *TcSPL5i*-MRE are conserved with alignment of presumed *SPL* orthologues of *Arabidopsis* and *Populous* [12, 33]*. TcSPL*1/3/8-MRE are fully conserved with MREs in the last exon of *SPL*s (*AtSPLs* and *PtSPL*s), and *TcSPL*2/4-MRE are highly conserved in the 3′ UTR of *SPL*s (Fig. 4c). *TcSPL1/3/8*-MRE and *TcSPL5*-MRE are in the same reading frame that participate in coding the conserved motif ALSLLS embedded in a non-conserved protein region (Fig. 4c). In particular, the *TcSPL5i*-MRE is poorly conserved in the 5′ half and encodes the non-conserved motif LQAPFC with different frames.

The four types of miR156-related MREs, specifically *TcSPL1/3/8-*MRE, *TcSPL*2/4-MRE, *TcSPL*5-MRE and *TcSPL5i*-MRE, were validated as target sites of TcmiR156 by dual-luciferase reporter (DLR) assay (Fig. 4d). The fluorescence intensity of the control group (i.e., the DLR vector and miRNA vector co-transformation) was set as 100%. Compared with the control group, fluorescence decreased in four MRE groups (i.e., MRE vectors and miRNA vector co-transformation) and recovered in four mutated MRE (mMRE) groups mismatched to 10th and 11th nucleotides of TcmiR156 (i.e., mMRE vectors and miRNA vector co-transformation) to different intensities. The results demonstrated that *TcSPL1/3/8-*MRE and *TcSPL5*-MRE were significantly depressed (< 2% fluorescence) by TcmiR156, and *TcSPL2/4-*MRE and *TcSPL5i*-MRE were depressed to a certain degree (< 40% fluorescence) by TcmiR156. The high fluorescence (> 50%) in the mMRE groups except *TcSPL*5-mMRE and the significant recovery (*P* < 0.01) in all mMRE vs MRE comparisons indicated that the 10th and 11th nucleotides are key sites for miR156-*TcSPL* interaction. The DLR assay suggests that *TcSPL1/2/3/4/5/8* were targeted by TcmiR156 in the manner of MRE interaction.

### Salt stress responses of miR156-targeted *TcSPLs*

**Fig. 5:**
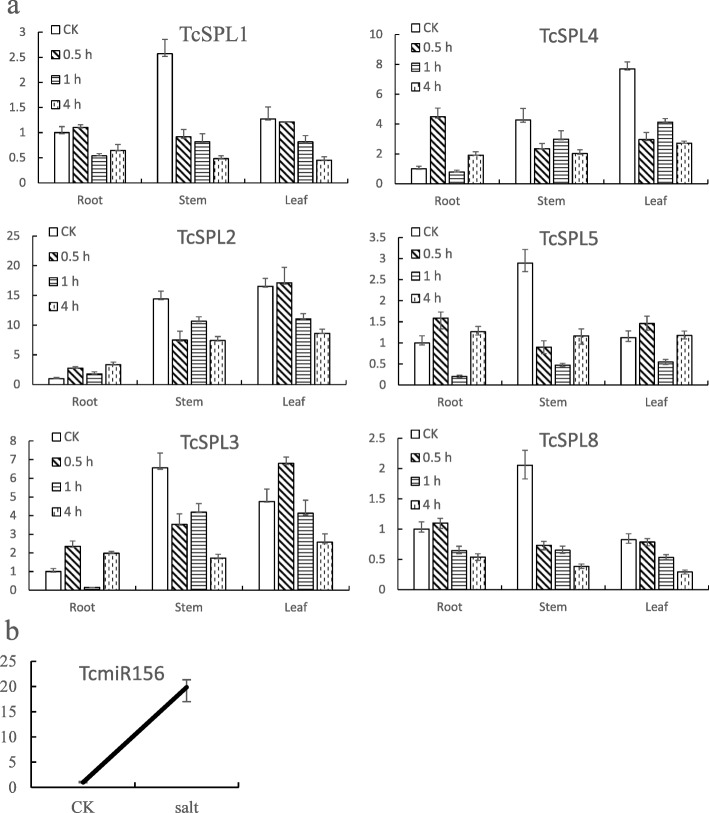
Expression profiles of the *SPL* genes of *Tamarix chinensis* (*TcSPLs)* targeted by miR156. **a** Relative expression levels of *TcSPL1–5, − 8* in 340 mM NaCl-treated roots, stems and leaves during 0.5 h, 1 h and 4 h. 0 h was used as the control group in each profile for the 2^-ΔΔCt^ relative quantification (RQ) method. Error bars represent RQ_max_ = 2^- (ΔΔCt-ΔCtSD)^ and RQ_min_ = 2^- (ΔΔCt + ΔCtSD)^ (*n* = 3). **b** Relative expression levels of miR156 in 340 mM NaCl-treated *Tamarix chinensis* for 1 h. 0 h was used as the control group, and the same RQ method was used

To explore the salt stress response patterns, expression levels of six miR156 target genes were examined in roots, stems and leaves during 2% NaCl treatment (Fig. 5a). All *TcSPLs* that were expressed in three tissues showed no tissue-specific expression pattern. The average expression level indicated that descending mRNA abundance of three tissues is as follows: stems, leaves and roots. In particular, the abundance of *SPL*s in stems is 2–14 times greater than that in roots under no salt stress, and *SPL*2 abundance differs significantly between roots and stems.

The salt stress response patterns of *SPL*s are similar in the same tissue and appeared to be two temporal expression patterns, that is, the shoots response pattern (tissues above ground) and the roots response pattern (tissues underground). For the shoot response pattern, *SPL*s were downregulated (0.5 h) and then stable (1 h and 4 h). Specifically, the more significant downregulation in stems (1.9–6.2-fold) than in leaves (1.8–2.8-fold) indicated that *SPL*s play important roles in the salt stress responses of stems. For the root response pattern, *TcSPLs* were ‘up-down-up’ -regulated with increasing salt stress time. In particular, *TcSPL3, TcSPL4,* and *TcSPL5* were significantly repressed in the 1 h salt-treated roots. Interestingly, *TcSPLs* were downregulated during 1 h NaCl treatment in all tissues, suggesting that 1 h is a key timing of the temporal expression patterns. Therefore, we further detected the expression level of TcmiR156 in 2% NaCl-treated *T. chinensis* (Fig. 5b). The results demonstrate that the salt stress responses of *TcSPLs* were negatively correlated with TcmiR156 expression level. All results indicated that *TcSPLs* under negative control of TcmiR156 play important roles in the salt stress responses.

## Discussion

### Conservation and divergence of *TcSPLs*

*SPL* families have been identified in green alga, moss, lycophyte, and seed plants, and their DNA-binding domain SBP-box is highly conserved throughout the plant kingdom [8]. Our study identified 14 *TcSPL* genes with conserved SBP-boxes (two incomplete SBP-boxes). The classification and names of 7 groups of the phylogenetic tree closely follow the groups hypothesis from *SPL* evolutionary study [8], except Group CR of green alga *SPL*s. In *SPL*s originating from land plants, the oldest Group I clusters all *SPL*s with C4-type zinc fingers, including *TcSPL*7, while other *TcSPLs* with C3H-type zinc fingers are grouped into younger Group IIa-f. In addition, the third residue divergence of the conserved DLS motif located in the N terminus of the Zn-1 structure was found in *TcSPL*7 and *TcSPL12*, indicating that *TcSPL12* appeared to be relatively early in origin. Unique motifs, including Motif 5 (unique to IIa) and Motifs 2, 6, 7 and 8 (unique to I and IIa), indicated that I and IIa may be associated with the functional diversity of the SBP family.

According to the number of *SPL* family members, *SPL* families of different plants can be roughly divided into two types: (1) canonical *SPL* family, such as *SPL* families of *Physcomitrella patens* [34], tomato [35], *Arabidopsis*, rice [36] with 13, 15, 16, 19 members, respectively, and (2) expansion of the *SPL* family due to gene duplication, such as *SPL* families of poplar [12] and maize [14] with 28 and 31 members, respectively. The *TcSPL* family, which has 14 members, should belong to the canonical family. Unlike one *PtSPL* paralogous pair or more that corresponded to one *AtSPL* gene, only 2 paralogous pairs (*TcSPL2/4* and *TcSPL3/5*) were predicted in *TcSPLs*, and the number of *TcSPLs* was largely the same as that of *AtSPL* in each group. These results indicate that *SPL* gene duplication of *Tamarix* and *Arabidopsis* remains linearly dependent and appears to occur before lineage divergence. The situation is similar in the canonical *SPL* family, such as rice, but opposite in the expansion *SPL* family, such as poplar with segmental duplication after lineage divergence.

### MRE within *TcSPLs*

The miRNA target sites within *SPL*s might exist before the divergence of moss and vascular plants [8]. In this study, almost all *SPL*s in IIc–IIf had miRNA target sites of miR156, and both the location and sequence of MREs are conserved in *TcSPLs*. This is consistent with the results from *Arabidopsis* and poplar *SPL*s [12, 33], suggesting that *SPL* posttranscriptional regulation is similar in plants. Six canonical MREs and a non-canonical *TcSPL5i*-MRE are predicted in *TcSPL1–5* and *− 8*. *TcSPL*1/3/5/8-MREs located in their last exon had higher conservation than *TcSPL*2/4-MREs located in their 3′-UTR. According to the evidence of higher conservation and conserved target motif ALSLLS, we suggest that the MRE located in the last exon might suffer more selective pressure than that in UTR. For *TcSPL5i*-MRE, its characterizations are non-canonical to all putative *SPL* orthologs, such as poor complementarity with miR156, lower pairing Gibbs free energy, first exon location and target motif. We speculate that *TcSPL5i*-MRE seems to be a specific translation inhibition site of the *SPL* family compared to *AtSPL3* [33]. The DLR assay proves the interaction of miR156 and MREs, and their interaction intensities are decreasing in the descending order *SPL*1/3/8-MRE > *SPL5* > *SPL2/4* > *SPL5i*, which is positively relative to their sequence complementarity. In addition, the DLR assay results showed that the 10-11th nucleotides are key sites for all canonical MREs, as well as the *TcSPL5i*-MRE, which appears to prove the uniqueness of translation inhibition.

### Response patterns of miR156-targeted *TcSPLs*

DGE analysis suggests that *TcSPLs* may play different roles in response to salt stress. The non-targeted *TcSPLs* except *TcSPL*7 are induced, while targeted *TcSPLs* are inhibited, by salt. In maize, 57% (11/19) of targeted *SPL*s are inhibited by salt or/and drought, and most non-targeted *SPL*s are induced [14], also indicating that expression patterns between targeted *SPL*s and non-targeted *SPL*s differ. In addition, highly abundant non-targeted *TcSPLs* (*TcSPL6, − 9, − 10*) belonging to IIa may be associated with many unique motifs identified in IIa.

Furthermore, we investigated the temporal expression pattern of targeted *TcSPLs* in different tissues to determine their salt stress response features. The targeted *TcSPLs* in leaves/stems remained stable after the quick downregulation showed a simple inhibition-response pattern to salt stress. The inhibitory effect was more dominant in stems. In roots, targeted *TcSPLs* show a complex response pattern ‘up-down-up’ –regulation. The upregulation during 0.5 h salt stress was consistent with DGE analysis. The downregulation during 1 h salt stress is consistent in DEG profiles in 2.5 h, suggesting a delay inhibition compared to stems/leaves. Together with the abundance recovered during 4 h salt stress, the targeted *TcSPLs* seem to be a valley-type (U curve) expression pattern to salt stress, and 1 h should be a key inverting time point. The significant negative correlation to stem-loop qPCR of TcmiR156 during 1 h salt stress confirmed this possibility. The negative expression trend is an indicator of regulation in miRNA-target profiles [37, 38]. The *TcSPLs* under the negative control of TcmiR156 play important roles in the salt stress responses. We suggest that TcmiR156 may play important roles in salt stress responses by negative control of *TcSPLs*. Many studies have shown the functional redundancy among *TcSPLs* in plant development regulation [39, 40]. Similar salt stress response patterns indicated that 6 miR156-targeted *TcSPLs* may be redundant in post-transcriptional regulation. Therefore, functional studies of miR156-targeted *TcSPLs* should employ experimental design that avoid potential obstacles due to redundancy.

## Conclusions

In this study, we conducted a systematic analysis of the *SPL* gene family in *T. chinensis*, examining 12 *TcSPL* genes clustered into 7 groups. The illustrated conserved motifs and MREs showed divergence among groups and similarity within each group. The DEG suggested potential different functions of miR156-targeted *SPL*s vs non-targeted *SPL*s in salt stress responses. MREs are conserved in location and sequence with the exception of *TcSPL5i*-MRE. The miR156-targeted *SPL*s are proved by DLR assay of MRE-miR156 interaction. The expression patterns of different tissues suggested that 1 h (salt stress time) could be a critical time point of miR156-targeted *SPL* salt stress responses. Our work provides a foundation for further post-transcriptional regulation studies of *SPL*s and would be helpful for determining the miR156/*SPL*-mediated molecular mechanisms underlying *T. chinensis* salt stress tolerance.

## Methods

### Plant materials

All materials were collected from the one-month-old seedings of the same *T. chinenses* ortet grown in campus of Nanjing Forestry University (Nanjing, Jiangsu, China). The roots of seedings were immersed in 340 mM NaCl solution for 0 h, 0.5 h, 1 h and 4 h. Roots, stems and leaves were cleaned after collection and immediately frozen in liquid nitrogen and stored at − 80 °C.

### Prediction and identification of *TcSPL* and source of other *SPLs*

Genes and protein sequences were obtained from the assembled transcripts of RNA-seq data [32]. By the hidden Markov model (HMM) prediction referring to SBP domain profile from Pfam (pfam03110, http://Pfam.sanger.ac.uk/), *TcSPL* transcripts were predicted. All candidate *TcSPL* genes further were identified in plant transcription factor database by the BLASTP searches and the sequences lacking SBP-boxes were rejected. Full cDNA sequences of *TcSPL1–5* were identified by Rapid Amplification of cDNA End (RACE) according to the manual of SMARTer RACE 5′/3′ Kit (Clotech, CA, USA) with slight improvement [41–43]. Predicted complete CDS of *TcSPL*6–10 were identified by sequencing of corresponding sequences amplification. *TcSPL11–14* are lack of complete CDS and their partial CDS or protein sequences were used for following analysis. *TcSPL13*, − 14 lack of full SBP-box were rejected in phylogenetic analysis. All sequences are listed Additional file 1: Table S1.

Additionally, Genes and protein sequences of *AtSPL*s and *PtSPL*s are downloaded from the *Arabidopsis* information resource database (http://www.Arabidopsis.org/) and Phytozome database (https://phytozome.jgi.doe.gov/), respectively (Additional file 1: Table S1).

### Sequence alignment and phylogenetic analysis

Multiple sequence alignment of the SBP-boxes of *TcSPLs*, *AtSPL*s and *PtSPL*s were obtained by alignment method MUSCLE. A phylogenetic analysis was performed using MEGA 7.0 [44] with the neighbor-joining method and 1000 bootstrap replicates.

### Motif analyses and MRE analyses of *SPL* genes

The amino acid sequence length, molecular weight and isoelectric point of the putive protein were calculated using the protparam program (http://web.expasy.org/protparam/). Motifs were identified using MEME program (http://meme-suite.org/tools/meme) with default parameters. The miR156-targeted *SPL* genes and MREs were predicted by psRNATarget (http://plantgrn. noble.org/psRNATarget/). Compare of MRE within *TcSPLs* with miR156 and conservation of MRE within *TcSPLs*, *AtSPL*s and *PtSPL*s were analyzed by multiple sequence alignment with software CLUSTAL X [45].

### Expression analysis of *TcSPL* targeted by TcmiR156

The total RNA extraction from different tissues of *T. chinensis* and cDNA synthesis was achieved using RNAprep Pure Plant Kit (DP441, Tiangen, Beijing, China) and PrimeScript™ RT Master Mix RT Master (RR036Q, Takara, Dalian, China) according to the manufacturer’s instructions. qRT-PCR analysis was performed in Viia 7 Real Time PCR System (ABI, California, USA) with saturated dye EvaGreen (Biotium, CA, USA) followed recommended qPCR Master Mix reaction mixture and amplification procedure. The gene-specific primers listed in Additional file 2: Table S2 were designed employing ExpressionSuite software (ABI, CA, USA). The relative expression levels were calculated using the 2^−∆∆Ct^ method based on reference gene TcTIF (Additional file 2: Table S2).

### Expression analysis of TcmiR156

Each 1 μg total RNA of mixed tissues of *T. chinensis* under no stress and under 1 h NaCl stress was reverse-transcribed using the *PrimeScript* RT reagent kit (Takara, Dalian, China) with additional stem-loop primer [46] of TcmiR156 (Additional file 2: Table S2). The qRT-PCR and analysis method were same as mentioned above.

### The dual-luciferase reporter (DLR) assay of MRE

The dual-reporter refers to the simultaneous expression and measurement of two individual reporter enzymes within a single system. In the DLR assay, the activities of firefly (*Photinus pyralis*) and Renilla (*Renilla reniformis*) luciferases are measured sequentially from a single sample. P2GW7 vector with TcmiR156 insertion was used as effector for transient overexpression of 20-nt mature miR156. 35SGLO vector was set as reporter by inserting MRE in 3’UTR of firefly luciferase. After co-transformation of effector and reporter into *Populus* protoplast, the fluorescence intensity generated from firefly luciferase and Renilla luciferase were automatically detected in Glomax-96 microplate luminometer with four replicates of each DLR assay. The luciferin substrate was prepared employing the Dual-Luciferase Reporter Assay Kit (Promega, WI, USA).

## Additional files

Additional file 1: **Table S1.** Source and sequences of *SPLs* for the phylogenetic analysis. (XLSX 24 kb)

Additional file 2: **Table S2.** Primers for qRT-PCR. (XLSX 9 kb)

Additional file 3: **Figure S1.** Gene structures of *TcSPL1–5, − 8*. (PDF 48 kb)

## Data Availability

All *TcSPL* cDNA sequences and primers are available in Additional files.

## Abbreviations

CDS: Coding sequence
DGE: Digital gene expression profiles
DLR: Dual-luciferase reporter
HMM: Hidden Markov model
ML: Maximum likelihood method
mMRE: Mutated MRE sequence
MRE: miRNA response element
NLS: Nuclear localization signal
pI: Isoelectric point
RACE: Rapid Amplification of cDNA Ends
ROS: Reactive oxygen species
SBP: SQUAMOSA promoter-binding
SOS: Salt overly sensitive
SPL: SBP protein-like proteins
SPL-MRE: mRNA cleavage MRE within SPL
TcSPLi-MRE: Translation inhibition MRE within SPL
UTR: Untranslated region
