# Supplementary figures and images for: Roles of the SPL gene family and miR156 in the salt stress responses of tamarisk (Tamarix chinensis)
Source: BMC Plant Biol. 2019 Aug 22;19:370. doi: 10.1186/s12870-019-1977-6 (PMC6704519; doi:10.1186/s12870-019-1977-6)

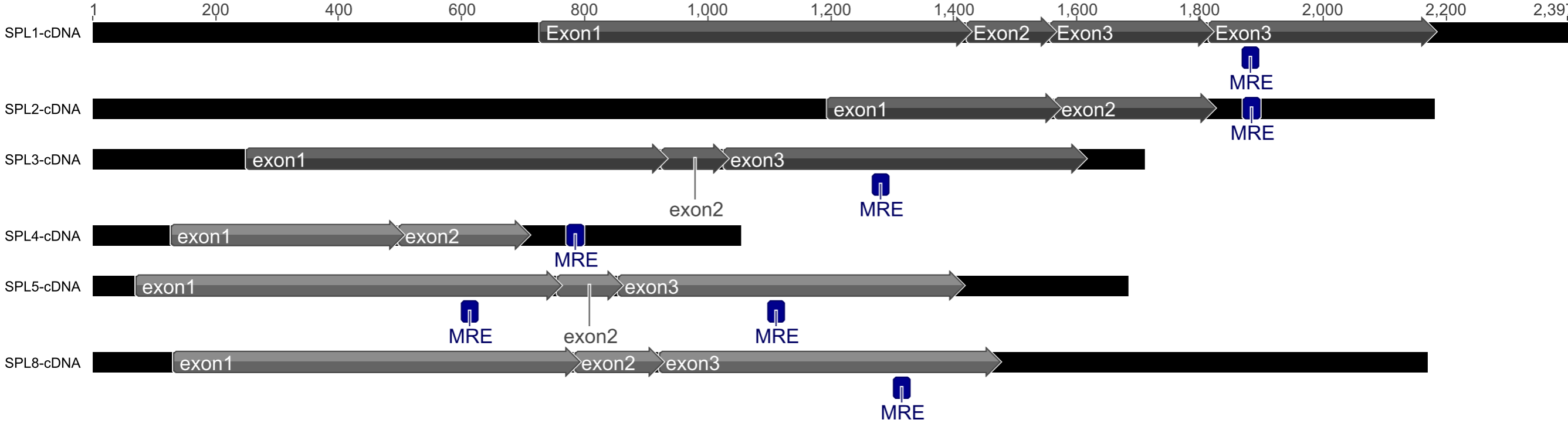

Supplement: Supplementary file 3 — Figure S1. Gene structures of TcSPL1–5, − 8. (PDF 48 kb) [file 12870_2019_1977_MOESM3_ESM.pdf]
